# Prevalence and factors associated with stunting and thinness among adolescent students in Northern Ethiopia: a comparison to World Health Organization standards

**DOI:** 10.1186/s13690-015-0093-9

**Published:** 2015-10-28

**Authors:** Yohannes Adama Melaku, Gordon Alexander Zello, Tiffany K. Gill, Robert J. Adams, Zumin Shi

**Affiliations:** 1 School of Public Health, College of Health Sciences, Mekelle University, Mekelle, Ethiopia; 2 College of Pharmacy and Nutrition, University of Saskatchewan, Saskatoon, Canada; 3 School of Medicine, The University of Adelaide, Adelaide, South Australia Australia

**Keywords:** Adolescent, Student, Ethiopia, Undernutrition, WHO standards

## Abstract

**Background:**

Adolescence is last chance for curbing the consequences of malnutrition and breaking the intergenerational cycle of malnutrition and poor health. This study aimed to assess the prevalence and the factors associated with stunting and thinness among in-school adolescents in northern Ethiopia using the 2006 World Health Organization (WHO) standards.

**Methods:**

In-school adolescents (*n* = 348, 10–19 years old) were randomly selected to participate in this cross-sectional study. Anthropometric measurements were carried out to determine the proportion of adolescents who were stunted (height-for-age < −2 Standard Deviation (SD)) and thin (body-mass-index-for-age < −2 SD). *T*-test was employed to evaluate mean weight and height differences between groups. Pearson chi-square, chi-square trend and Fisher’s exact tests were used to explore the crude association of categorical outcome variables and associated factors. Crude and adjusted associations between the outcome variables (stunting and thinness) and independent variables (socio-demographic, eating behavior and sanitation) were also determined using logistic regression. Stata version 11.1 was used to analyze the data.

**Results:**

The height of the adolescents was 147.6 ± 11.2 cm (mean ± SD) and weight was37.2 ± 9.5 kg. The mean Z-scores of height-for-age and body-mass-index (BMI)-for-age of adolescents were −1.49 and −1.29, respectively. The prevalence of stunting and thinness among adolescents was 28.5 % (boys = 37.7 %; girls = 21.2 %; *P* = 0.001) and 26.1 % (boys = 32.4; girls = 21.6 %; *p* = 0.017), respectively. Adolescents in 13–15 year old age group (Adjusted Odds ratio (AOR) = 2.23; 95 % CI: 1.22, 4.08), boys (AOR = 2.53; 95 % CI: 1.52, 4.21) and rural residents (AOR = 2.15; 95 % CI: 1.20, 3.86) had significantly higher odds of being stunted compared to their counterparts. Furthermore, boys had higher (AOR = 1.97; 95 % CI: 1.19, 3.25) odds of being thin compared to girls. Compared to those 10 to 12 years of age, adolescents in 16 to 19 years of age were 53 % (AOR = 0.47; 95 % CI: 0.23, 0.95) less likely to be thin.

**Conclusions:**

Undernutrition is widely prevalent among adolescents in northern Ethiopia. Sex, age and area of residence significantly associated with adolescent undernutrition. The study underlines the need for nutrition interventions targeting rural and boy adolescents.

## Background

According to the World Health Organization (WHO) adolescence is defined as10-19 years of age [1]. Adolescents represent approximately 20 % of the world’s population and most (~84 %)are living in developing or emerging countries [2]. In Ethiopia, 20-26 % of the population was represented by adolescents [3]. Adolescence is a period characterized by important biological, physical, psychological and social changes [4] and an active growth phase [5]. Throughout this period, risk of nutrition inadequacies and other health issues are of concern due to rapid growth in stature, muscle mass and fat mass. During the peak of the adolescent growth spurt, some dietary requirements are also as high as or higher than in other age groups [6]. Adequate nutrition and health are essential as adolescents gain up to 50 % of their adult weight, more than 20 % of their adult height, and 50 % of their adult skeletal mass during these years [7].

Despite the economic growth observed in developing countries, malnutrition and particularly undernutrition is still highly prevalent [8–12] though a growing prevalence of obesity is also being observed in some of these countries [8, 13]. Ethiopia is not an exception. A study in northern Ethiopia reported high levels of stunting (26.5 %) and thinness (58.3 %) [12]. On the other hand, increased prevalence (8.5 %) of overweight/obesity was reported among adolescents in a city (Addis Ababa) [14]. This double-burden of malnutrition in children and adolescents adversely affects their intellectual development [15–19], school attendance [20], growth [19], health [21], academic performance and social skills [15, 17, 19]. For instance, a longitudinal study in southwest Ethiopia demonstrated the positive relationship between adolescent food insecurity and absenteeism and low success in schooling [22].

Poor nutrition status among adolescents is also an important determinant of poor health outcomes. Undernutrition has far reaching consequences, especially in girls. If their nutritional needs are not met, they have high risk of mortality as a result of pregnancy and childbirth and they are more likely to give birth to low birth weight infants [23–25]. Furthermore, in all adolescents, short stature resulting from chronic undernutrition is associated with reduced lean body mass and deficiencies in muscular strength and working capacity [26]. Thus, one approach to break the intergenerational cycle of malnutrition and poor health is to improve the nutrition of adolescents; otherwise, the vicious cycle will continue.

In Ethiopia, although childhood stunting is highly prevalent in general and in the region (Tigray) where this study was conducted in particular [10], data on adolescents are scarce. The few existing studies have reported varying levels of undernutrition among adolescents between 2009 and 2014. A national level survey on adolescent girls reported that 23 % and 14 % of adolescent girls were stunted and thin (as defined by WHO 2006 standard; Z-score < −2), respectively [10]. In Addis Ababa, the capital of Ethiopia, 7.2 % and 6.2 % of adolescents were found to be stunted and thin, respectively [14]. A study conducted among adolescent girls in rural communities of northern Ethiopia showed that the prevalence of stunting and thinness were 26.5 % and 58.3 %, respectively [12]. Another study conducted in Ambo town showed that underweight was prevalent in boys than girls (29.8 % versus 24.6 %) with total prevalence of 27.5 % [27].

To assess the nutrition status of adolescents, the WHO currently recommends using BMI-for-age and height-for-age [28, 29]. The WHO growth standard for adolescents also uses thinness (low body-mass-index (BMI)-for-age (BAZ)) and stunting (low height-for-age (HAZ)) [28, 29]. The main difference between these two indicators is that the former is a result of mainly acute (short-term) nutrient deficiency (specifically macronutrients) whereas the later shows chronic (long-term) deficiency. Previous studies have used these standards to evaluate the nutrition status of adolescents and to compare prevalence of thinness and stunting in different settings [30–33].

Investigating growth, health and nutrition status during the adolescence stage of development, in addition to infancy and childhood is important. However, there are few studies that investigate stunting and thinness during adolescence in Ethiopia [12, 14, 34, 35] and there is only one study that has been conducted in northern Ethiopia with a focus on adolescent girls [12].

The main aim of this study was, therefore, to assess the prevalence and the factors associated with stunting and thinness among in-school adolescents (both boys and girls) in northern Ethiopia using the WHO standards [36]. Data obtained from this study are relevant to both governmental and non-governmental organizations interested in improving the health and nutrition of an important and often neglected segment of the population in developing and emerging countries.

## Methods

### Study design, setting, sample size determination and sampling technique

The study design was a school-based, cross-sectional study measuring the prevalence and factors associated with stunting and thinness among adolescents in a town in northern Ethiopia. The study was carried out from February to March, 2014.

The sample size was determined using a single population proportion formula, with the following assumptions: Maximum allowable error was set at 4 %; the proportion of stunted Ethiopian adolescents at 16 % [35]; Z statistic of 1.96; and estimated non-response rate of10%. A sample size of 356 was determined; however, 8 students (3 boys and 5girls) declined to participate in the study, resulting in a final sample size of 348.

The study was conducted in Wukro in the Tigray Region of northern Ethiopia. Wukro is located approximately 829 kilometer (km) north of Addis Ababa and 45 km north of Mekelle (capital of the Tigray region). The total population is estimated to be more than 40,000. Six primary (Zikre-semaetat, Ksanet, Millennium, Kidus-Yossef, Bethntset and Selam) and two secondary (Megabit-30 and Wukro) schools were present in Wukro at the time of the study. In the academic year of 2013/2014, 10,073 adolescents (10–19 years of age) were attending school encompassing grades 5 to 12. The WHO definition of the age range of adolescents (10–19 years) was used as the inclusion criteria for the study [1].

The number of study participants in both the primary and secondary schools was determined proportionally to population size. Three schools, namely Ksanet, Millennium and Selam, were selected using simple random sampling from the six primary schools in Wukro. Both of the secondary schools (i.e. Megabit 30 and Wukro) in Wukro were included. Primary and secondary schools were represented by 198 and 150 students, respectively.

**Fig. 1:**
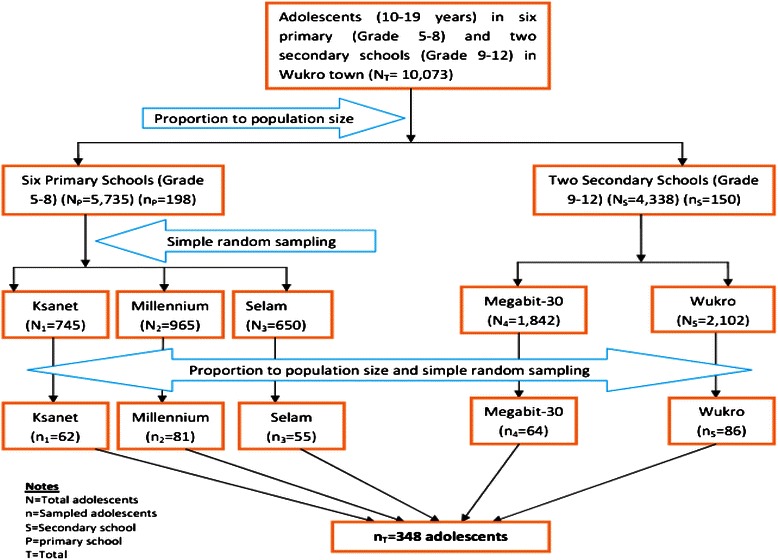
Schematic presentation of participant enrollment

Sampling frames (list of students between 10–19 years) were obtained from each school’s administrators. Study participants from each school were then selected using a simple random sampling technique after allocating the number of study participants using proportion to population size. Room teachers (i.e. those responsible for supervision of students) assisted in identifying study participants. A schematic representation of participant selection is shown in Fig. 1.

### Measurements

A pretested, self-administered, structured questionnaire (including both open-ended and closed–ended questions) was used in the study. The pretest was conducted among 38 students (10 % of the sample) in schools that were found in the nearby town. The questionnaire was developed by reviewing previous literature [11, 12, 14, 27]. The English version of the structured questionnaire was translated into the local language, “Tigrigna”. The questionnaire focused on socio-demographic characteristics (sex, age, fathers’ and mothers’ education, usual place of residence, fathers’ occupation, and number of family members), sanitation (functional latrine availability), eating behavior and anthropometric measurements. Each participant was asked about frequency of diet and type of foods eaten per day and week. Staple foods such as “Teff” (a cereal) with “Wot” (primarily prepared from legumes), wheat and others, vegetables, fruits and meat were included in the questionnaire. The presence of a functional latrine and water source for the household were also assessed. Furthermore, age differences of adolescents from their younger and older siblings were considered as a factor in the analysis in order to look the effect of age difference of siblings on adolescent nutrition status. Heights (to the nearest 0.1 cm) and weights (to the nearest 0.1 kg) of adolescents were measured using a stadiometer and balance beam scale (SECA, Hannover Germany), respectively. The height of each adolescent was measured without shoes. Weight was also measured without jackets or coats, shoes and additional clothing. Data collectors, who were university students, were trained on the contents of the questionnaire, procedures for data collection, data accuracy and completeness. Field work was conducted under close supervision of the principal investigators.

In each school, an official communication was used to identify a suitable time for data collection. Before completing the questionnaire, respondents were given a clear introduction explaining the purpose and objectives of the study. Data were collected during a single day in each participating school in the absence of class teachers, and efforts were made to ensure maximum comfort and privacy for the participants. Students were not allowed to discuss their questionnaire responses with each other, both for reasons of privacy and to avoid shared responses.

### Data management, quality control and analysis

All possible actions to ensure quality of the data were performed before, during and after data collection. Training of data collectors and supervisors, pretesting of the questionnaire, and standardization of measuring scales of weight and height were undertaken. During data collection, consistency and completeness of questionnaires were checked by supervisors who had Bachelor degrees in health sciences. After data collection, unique codes (numbers) were provided for the questionnaire to facilitate cleaning of data whenever errors were detected. Data were entered into Statistical Package for Social Sciences (SPSS) version 20 (*SPSS*, IBM, New York) and WHO Anthro-Plus. They were then exported to Stata version 11.1 (Stata Corporation, College Station, TX, USA) for analysis.

Descriptive analysis such as mean, median, standard deviation (SD) and inter-quartile range (IQR) were used to describe characteristics of the study participants. Anthropometric analysis determined the proportion of adolescents who were stunted (HAZ < =2SD) and thin (BAZ < −2 SD) [29]. To evaluate mean weight and height differences between boys and girls, student *T*-test was used. In addition, *χ*^2^, *χ*^2^trend and Fisher’s exact tests were used to investigate the relationship of categorical outcome variables and associated factors.

The association between the outcome variables (i.e. stunting and thinness) and independent variables were first analyzed using bivariable logistic regression model. Covariates having p-value <0.25 were retained and entered to the multivariable logistic regression analysis [37]. A p-value <0.05 was considered as a cut-off point for an independent variable to be significantly associated with the outcome. In the analyses, Hosmer-Lemeshow *χ*^2^ and Variance Inflation Factor (VIF) assessed the goodness-of-fit of the models and multi-co-linearity, respectively. All p-values of Hosmer-Lemeshow and VIF were greater than 0.05 and less than 10 %, respectively.

### Ethical considerations

Ethical approval and an ethical clearance letter were obtained from the Institutional Review Board of Mekelle University. The education offices of Wukro, as well as school directors in Wukro, were informed through formal letters. The majority of students were less than 18 years and were unable to give consent. Considering this and since the questionnaire didn’t contain sensitive issues, thorough discussions were undertaken with the school directors regarding the purpose and the contents of the data collection tool, and permission was obtained to conduct the study. Respondents were given assurances about the privacy and confidentiality of their responses. Informed oral consent was obtained from each participant to ensure their willingness to participate, and all were informed that they had the right to not participate or could withdraw from the study at any time.

## Results

### Socio-demographic characteristics of adolescents in relation to nutrition status

More than half of the study participants were girls and the median age of both sexes was 14 years. Two-thirds of the participants resided in urban areas. Nine out of ten of the participants identified as Orthodox Christians. The literacy rate of fathers and mothers were found to be 63.5 % and 55.2 %, respectively (Table 1).

**Table 1:** Socio-demographic characteristics of adolescents categorized by nutrition status (i.e. stunted and thin), northern Ethiopia, 2014

Variables	Category	Total	Stunted (<−2 SD) *n* (%)	*P*-value*	Thin (<−2 SD) *n* (%)	*P*-value*
Sex	Boys	154 (44.3)	58 (37.7)	0.001	50 (32.5)	0.017
	Girls	194 (55.7)	41 (21.1)		41 (21.1)	
Age in years	10-12	120 (34.5)	25 (20.8)	0.002	35 (29.2)	0.036
	13-15	135 (38.8)	53 (39.3)		41 (30.4)	
	16-19	93 (26.7)	21 (22.6)		15 (16.1)	
Median age (IQR)	14 (IQR = 12,16)					
Age difference from elder sibling	I am the 1^st^child for my mother	77 (22.1)	17 (22.1)	0.401	16 (20.8)	0.478
	<=2 years	94 (27.0)	28 (29.8)		26 (27.7	
	>2 years	177 (50.9)	54 (30.5)		49 (27.7)	
Age difference from younger sibling	I am the last child for my mother	55 (15.8)	11 (20.0)	0.256	16 (29.1)	0.383
	<=2 years	92 (26.4)	30 (32.6)		28 (30.4)	
	>2 years	201 (57.8)	58 (28.9)		47 (23.4)	
Usual place of Residence	Urban	217 (62.4)	51 (23.5)	0.008	64 (29.5)	0.068
	Rural	131 (37.6)	48 (36.4)		27 (20.6)	
Religion	Orthodox	323 (92.8)	96 (27.7)	0.067**	82 (25.4)	0.245
	Muslim	25 (7.2)	3 (12.0)		9 (36.0)	
Education of father	Illiterate	127 (36.5)	43 (33.9)	0.090	28 (22.1)	0.187
	Literate	221 (63.5)	56 (25.3)		63 (28.5)	
Education of Mother	Illiterate	156 (44.8)	44 (28.2)	0.928	33 (21.2)	0.056
	Literate	192 (55.2)	55 (28.7)		58 (30.2)	
Occupation of father	Employed	150 (43.1)	28 (18.7)	0.002	40 (26.7)	0.069
	Farmer	131 (37.6)	48 (36.6)		27 (20.6)	
	Other	67 (19.3)	23 (34.3)		24 (35.8)	
Family size	<=4 members	98 (28.2)	18 (18.4)	0.009	24 (24.5)	0.645
	>4 members	249 (71.8)	81 (32.5)		67 (26.9)	

*P-value was determined using chi-square test except for **; **Fisher’s exact test

Stunting and thinness were significantly associated with sex. The overall prevalence of stunting among boys and girls were 37.7 % (95 % CI: 30.3 %, 45.5 %) and 21.2 % (15.8 %, 27.3 %) (*p* = 0.001), respectively. Likewise, the prevalence of thinness among boys and girls were 32.4 % (95 % CI: 25.4 %, 40.2 %) and 21.6 % (95 % CI: 15.8 %, 27.3 %) (*P* = 0.017), respectively (Table 1). Age was associated with stunting (*p* = 0.002) and thinness (*p* = 0.036). A higher percentage in rural area were stunted (*p* = 0.008) compared to those living in an urban area. Participants whose fathers were farmers had a higher prevalence of stunting than those with other occupations (*p* = 0.002). Larger families were associated with greater prevalence stunting in adolescents (*p* = 0.009). Stunting was found to be higher among families whose members were >4 (*p* = 0.009) (Table 1).

### Eating behavior and hygienic practices in relation to nutrition status

The majority, (56.3 %) of adolescents consumed “Teff” with “Wot” as a daily staple in the diet and the frequency of consumption most commonly three or more times per day (83.6 %). Vegetables (91.4 %) and fruits (89.1 %) were also consumed at least once daily. Three-quarters (75.5 %) of adolescents ate meat at least once per week. The majority of the adolescents reported that they used piped water and had a functional latrine at their home (Table 2).

**Table 2:** Sanitation practices and eating behavior of adolescents categorized by nutrition status (i.e. stunted and thin), northern Ethiopia, 2014

Variables	Category	n (%)	Stunted	*P*-value*	Thin	*P*-value*
			*n* (%)		*n* (%)	
Kind of food always eaten at home	“Teff“with “Wot”	196 (56.3)	46 (23.5)	0.051	53 (27.0)	0.888
	Wheat	122 (35.1)	41 (33.6)		31 (25.4)	
	Others	30 (8.6)	12 (40.0)		7 (23.3)	
Number of feedings per day	1-2 times	57 (16.4)	16 (28.1)	0.945	15 (26.3)	0.975
	> = 3 times	191 (83.6)	83 (28.5)		76 (26.1)	
Eat vegetable at least once per day	Yes	317 (91.4)	88 (27.8)	0.302	84 (26.5)	0.706
	No	30 (8.7)	11 (36.7)		7 (23.3)	
Eat fruits at least once per day	Yes	309 (89.1)	85 (27.5)	0.229	81 (26.2)	0.989
	No	38 (11.0)	14 (36.8)		10 (26.3)	
Eat meat at least once per week	Yes	262 (75.5)	70 (26.7)	0.189	63 (24.0)	0.105
	No	85 (24.5)	29 (34.1)		28 (33.0)	
Source of water	Pipe water	309 (88.8)	84 (27.2)	0.141	81 (26.2)	0.939
	Other (well, river….)	29 (11.2)	15 (38.5)		10 (25.6)	
Presence of functional latrine at home	Yes	310 (89.1)	90 (29.0)	0.490	79 (25.5)	0.420
	No	38 (10.9)	9 (23.7)		12 (31.6)	

*P-value was determined using chi-square test

### Anthropometric measurements

The mean ± SD overall height of the participants was 147.6 ± 11.2 cm (95 % CI: 146.5, 148.8) and weight was 37.2 ± 9.5 kg (95 % CI: 36.2, 38.2). The mean heights of boys and girls were 147.6 ± 13.40 cm (95 % CI: 145.5, 149.8) and 147.7 ± 9.10 cm (95 % CI: 146.4, 148.9) respectively with no statistical difference between the two groups (*p* = 0.967). Similarly, the mean weights of boy and girl adolescents were 35.9 ± 9.72Kg (95 % CI: 34.4, 37.5) and 38.3 ± 9.22Kg (95 % CI: 37.0, 39.6) respectively with statistical difference between the two groups (*P* = 0.023) (data not shown).

**Fig. 2:**
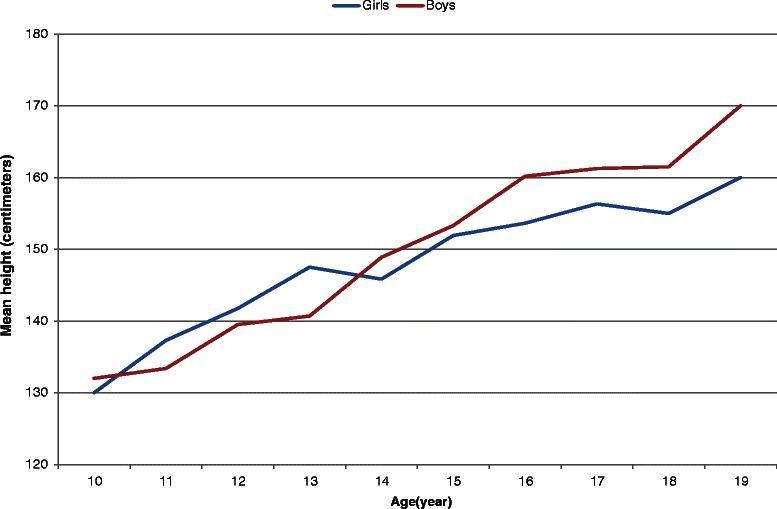
Mean height of boy and girl adolescents by age, northern Ethiopia, 2014

The mean height of boy and girl adolescents in relation to age is shown in Fig. 2. Boys were taller at 10 years of age while between the ages of 11 and 13 years, boys were shorter. At the age of 14 years, girls were shorter than boys.

**Fig. 3:**
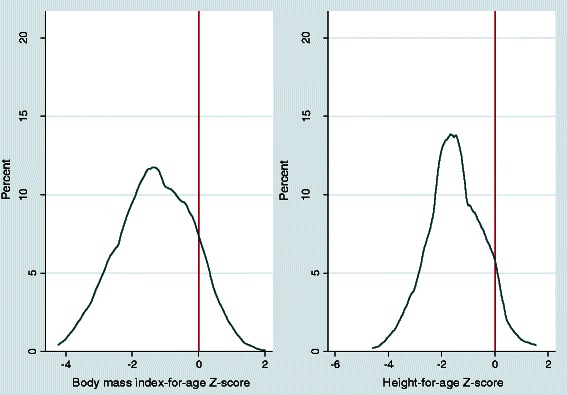
Kernel density plots of Body-mass-index-for-age and Height-for-age Z-scores for all adolescents, northern Ethiopia, 2014

The mean Z-scores of height-for-age and BMI-for-age of all adolescents were −1.49 (95 % CI:-1.60, −1.39) and −1.29 (95 % CI:-1.41,-1.18), respectively. The mean Z-scores of height-for-age among boys and girls were −1.68 (95 % CI: −1.85,-1.50) and-1.34 (−1.48,-1.20) respectively, which are significantly different (*p* = 0.0028). Similarly, the mean Z-scores of BMI-for-age among boys and girls were −1.59 (95 % CI:-1.75, −1.43) and −1.06 (95 % CI: −1.22, −0.89), respectively (*p* < 0.0001). Generally, Z-scores of BMI-for–age and height-for-age for all adolescents and by sex were found to be below the 2006 WHO standards and there were almost no obese cases (Fig. 3).

### Prevalence of stunting and thinness

The prevalence of stunting and thinness among adolescents was 28.5 % (95 % CI: 23.9 %, 33.4 %) and 26.1 % (95 % CI: 21.7 %, 31.0 %), respectively. The prevalence of severe stunting (HAZ < −3SD) and thinness (BAZ < −3SD) was 8.1 % and 7.1 %, respectively. The trend of stunting (*p* = 0.711) and thinness (*p* = 0.229) across age was not significant (data not shown).

### Factors associated with stunting and thinness

In bivariable logistic regression, sex, age, residence, family size, education of father, type of food usually eaten were found to be significantly associated with stunting. However, in multivariable analysis only sex, age, residence and fathers’ occupation were significantly associated with stunting.

Boy adolescents had 2.53 times higher odds of stunting (AOR = 2.53; 95 % CI: 1.52, 4.21) compared to girls. Adolescents age 13–15 years had 2.23 times higher odds of being stunted (AOR = 2.23; 95 % CI: 1.22, 4.08) compared to those aged 10 to 12, Rural residents had 2.15 times higher odds of being stunted (AOR = 2.15; 95 % CI: 1.20, 3.86) than their counterparts. Similarly, a marginal association of the occupation of the father and stunting was detected as adolescents whose fathers’ occupation was farmers had 2 times higher odds of being stunted (AOR = 2.00; 95 % CI: 1.00, 4.00) compared to adolescents whose fathers were employed (Table 3).

**Table 3:** Factors associated with stunting and thinness among adolescents in northern Ethiopia, 2014

		Stunting			Thinness		
Variable	Category	COR (95 % CI)	AOR (95 % CI)	*P*-value*	COR (95 % CI)	AOR (95 % CI)	*P*-value*
Sex	Boy	2.25 (1.40, 3.62)	2.53 (1.52, 4.21)	<0.001	1.79 (1.11, 2.91)	1.97 (1.19,3.25)	0.008
	Girl	1.00	1.00		1.00	1.00	
Age	10-12 years	1.00	1.00		1.00	1.00	
	13-15 years	2.46 (1. 40, 4.30)	2.23 (1.22, 4.08)	0.009	1.06 (0.62, 1.81)	1.11 (0.63,1.97)	0.712
	16-19 years	1.11 (0.58,2.14)	0.83 (0.41, 1.67)	0.608	0.47 (0.24, 0.92)	0.47 (0.23, 0.95)	0.035
Residence	Urban	1.00	1.00		1.00	1.00	
	Rural	1.87 (1.16, 3.01)	2.15 (1.20, 3.86)	0.01	0.62 (0.37, 1.04)	0.73 (0.40,1.31)	0.286
Family size	<=4 members	1.00	1.00		1.00	1.00	
	>4 members	2.14 (1.20, 3.81)	1.75 (0.95, 3.21)	0.071	1.14 (0.66, 1.95)	1.15 (0.65, 2.01)	0.639
Occupation of father	Employed	1.00	1.00		1.00	1.00	
	Farmer	2.50 (1.45, 4.30)	1.34 (0.99, 2.34)	0.234	0.71 (0.41, 1.25)	Omitted	
	Other	2.26 (1.18, 4.33)	2.00 (1.00,4.00)	0.05	1.54 (0.83, 2.84)	1.53 (0.80, 2.91)	0.196
Type of food usually eaten (staple)	*"Teff" with “Wot”*	1.00	1.00		1.00	1.00	
	Wheat	1.67 (1.01, 2.76)	1.34 (0.77, 2.31)	0.297	0.92 (0.55, 1.54)	1.00 (0.58, 1.73)	1.000
	Other	2.17 (0.98, 4.85)	1.66 (0.70, 3.95)	0.249	0.82 (0.33, 2.03)	0.85 (0.33, 2.19)	

*COR*-Crude Odds Ratio; *AOR*-Adjusted Odds Ratio; *CI*-Confidence Interval; *Z-test

In multivariable analysis, after adjusting for residence, family size, father’s education and type of food usually consumed and sex and age, only sex and age were found to be significantly associated with thinness. Boys had almost 2 times higher odds of being thin (AOR = 1.97; 95 % CI: 1.19, 3.25) compared to girls. Compared to those 10 to 12 years of age, adolescents aged 16 to 19 years were 53 % less likely to be thin (AOR = 0.47; 95 % CI: 0.23, 0.95) (Table 3).

## Discussion

### Prevalence of undernutrition

The present study shows that the extent of undernutrition among adolescents in northern Ethiopia is high. The prevalence of stunting in adolescents was 28.5 % with a significant difference between boys and girls (37.7 % and 21.2 %, respectively). Previous studies in adolescent Ethiopians reported much lower levels of stunting. These include studies in the Jimma zone (16 %) [35] and Addis Ababa (7.2 %) [14]. Nonetheless, in northern Ethiopia, the prevalence of childhood chronic malnutrition is very high [10] which may have impact on level of adolescent stunting. A number of studies in other African countries including Burkina Faso (8.8 % [38], 12 % [39]) and Chad (18.7 %) [40] have reported a lower prevalence of stunting. However, a high prevalence of stunting in adolescent girls has been reported in Bangladesh (32 %) [41], northern Ethiopia (26.5 %) [12] and Seychelles (23 %) [42]. In contrast to these countries, In China, a study reported that the prevalence of stunting is much lower to the level that it is no longer a nutritional problem of public health importance [43].

Stunting is an indicator of chronic malnutrition, and at school age, it may reflect malnutrition during the first years of life [9]. Adult short stature results from nutritional deficit at the different phases of growth rather than just in a single phase [44]. Childhood stunting is highly prevalent in Ethiopia [10, 45, 46] and the last chance for curbing the consequences of malnutrition on height is the adolescence period. There is a potential that children who suffered childhood stunting could achieve catch-up growth during adolescence [47, 48] although it is usually incomplete [48].

In the current study, the prevalence of thinness was 26.1 %. A similar level of thinness was found in Seychelles (27.7 %) [42]. Another study in Ethiopia, in the Jimma zone, has reported a much higher level (80.8 %) of thinness prevalence among adolescents [35]. However, other studies in Ethiopia (6.2 %) [14] and Burkina Faso (13.7 % [38], 8.0 % [39]) have reported much lower prevalence. In our study, the prevalence of thinness among boys was significantly higher than girls (32.4 % and 21.6 %, respectively). Similarly, in Addis Ababa, the prevalence of thinness among boys was significantly higher than girls (9.8 % and 2.6 %; *p* < 0.01, respectively) [14]. The prevalence of thinness among girls in our study was lower than studies in Northern Ethiopia (58.3 %) [12] and Bangladesh (26 %) [41]. On the other hand, our study found higher level of thinness among girls compared to studies in Seychelles (14 %) [42], Iran (10.1 %) [49], Turkey (11.1 %) [50], Qatar (5.8 %) [51] and China (5.2 %) [43].

In the current study, the mean heights of boy and girl adolescents were147.6 and 147.7 cm, which were not significantly different (*p* = 0.967). A mean height (147 cm) among girl adolescents has previously been reported in northern Ethiopia [12]. In this study, the mean weights of boy and girl adolescents were 35.9 kg and 38.3 kg which were significantly different. A mean weight of 34.6 kg of female adolescents was previously reported in northern Ethiopia [12] but in contrast to our study, a study conducted in Addis Ababa found boys to be taller and heavier by 10 cm and 4 kgs, respectively [14]. Again, this may be due to the socio-economic environment, and differences between rural and urban settings in Ethiopia.

In the current study, the mean Z-scores of height-for-age and BMI-for-age of all adolescents were −1.49 and −1.29, respectively. In addition, there were no obese cases in the study. In Addis Ababa, Z-scores of height-for-age and BMI-for-age of the adolescents were, −0.72 and −0.48, respectively [14]. In our study, the mean Z-scores of height-for-age among boys and girls were −1.68 and −1.34 respectively which are significantly different (*p* = 0.0028). In another study in northern Ethiopia, a mean Z-score of height-for-age among girls was reported to be −1.5 [12] which is similar to what our study found. In our study, mean Z-scores of BMI-for-age among boys and girls were −1.59 and −1.06 (*p* < 0.0001), respectively. Consistent with our results, a study reported that mean Z-score of BMI-for-age was significantly higher in girls than boys [14]. Overall, our anthropometric data indicated adolescents living in a town which has both rural and urban residents had poorer nutrition status than those that live in the larger capital city of Addis Ababa. Nutritional Policies and programs against adolescent undernutrition should give focus and priority for rural areas where the problem is prevalent. These interventions are also equally important for small towns and peri-urban areas.

Globally, we found that Z-scores of height-for-age and BMI-for-age for all adolescents and by sex were found to be below the 2006 WHO standards. Other studies in Ethiopia have reported similar findings [12, 14]. The reason for scoring lower means of BAZ and HAZ compared to the WHO growth standard are not surprising as Ethiopia is considered a developing and/or emerging country where poor dieting habits and insufficient food intake remains a concern.

### Factors associated with undernutrition

In this study, adolescent boys had 2.5 times higher odds of stunting compared to girls. This finding is consistent with studies in south-west Ethiopia and Romania [26, 52]. However, opposite finding has been reported in Extremadura (Spain) [53]. Compared to those 10 to 12 years of age, adolescents 13–15 years of age had 2.2 times higher odds of being stunted. This finding is consistent with studies in Ethiopia, Burkina Faso and Bangladesh [12, 38, 41, 54] which demonstrated varying prevalence of stunting among different age groups. Rural residents had 2.2 times higher odds of being stunted than their urban counterparts. Similar to our result, studies in Ethiopia and western Africa [27, 35, 38, 55–58] have reported the same findings.

Boys had almost 2 times higher odds of being thin compared to girls which is similar with findings in Ethiopia and Romania [12, 35, 52]. In the current study, adolescents aged 16 to 19 years were 53 % less likely to be thin compared to those aged 10 to 12 years. Association between age and thinness were reported in studies conducted in Ethiopia [12] and Bangladesh [41]. In Ethiopia, national nutritional programs and interventions had special interest and focus on adolescent girls [59, 60]. However, the current study shows that, compared to girls, boy adolescents are significantly being affected by undernutrition. Therefore, nutritional programs and interventions should also give at least equal attention to boys.

**Box 1:** Key intervention recommendations

• In addition to children, nutrition interventions should consider adolescents as major target groups.	
• Despite the existing focus for girl adolescents, it is equally important to address nutrition problems of their counterparts.	
• Adolescents living in rural areas require more attention for nutrition interventions.	

### Limitations

We acknowledge some limitations in this study. Some variables such as the systematic measurement of nutritional knowledge, social and economic status, food security and dietary diversity score were not included. Furthermore, errors during anthropometric measurements are also possible and could lead to measurement bias. However, the data collectors were trained in anthropometric measurement procedures and they were also supervised during data collection. The study also did not collect information related to pubertal landmarks. Studies have shown that chronic under-nutrition can delay sexual maturity and the adolescent growth spurt. As a result, adjustments were not made for differences of ages of sexual maturation, which might confound comparisons between the survey and reference population [61, 62].

## Conclusions

Undernutrition is widely prevalent among adolescents in northern Ethiopia. Height-for-age and BMI-for-age scores of all adolescents were found to be below the 2006 WHO standards. Age and residency were found to be important factors associated with stunting. Age was also an important factor associated with thinness. Importantly, unlike previous studies in Ethiopia [12, 34], we found that boys were affected more by undernutrition (both by stunting and thinness) compared to girls. Programs to support nutrition interventions for adolescents could provide an opportunity for healthy transition from childhood to adulthood and could be an important step in breaking the intergenerational cycle of malnutrition. Recognizing the intergenerational effect of malnutrition and high prevalence of adolescent undernutrition, there is a clear need to give due attention to adolescent nutrition. In Ethiopia, strategies in addressing the undernutrition among adolescents (focusing on both boys and girls) are needed in addition to the existing efforts to combat child undernutrition. Further studies with more robust methods are required to explore dietary pattern and nutrient intake and their association with nutritional status of adolescents.
